# Critically ill patient mortality by age: long-term follow-up (CIMbA-LT)

**DOI:** 10.1186/s13613-023-01102-3

**Published:** 2023-02-11

**Authors:** João Gonçalves-Pereira, André Oliveira, Tatiana Vieira, Ana Rita Rodrigues, Maria João Pinto, Sara Pipa, Ana Martinho, Sofia Ribeiro, José-Artur Paiva

**Affiliations:** 1 Intensive Care Unit, Hospital Vila Franca de Xira, Estrada Carlos Lima Costa, N2, 2600-009 Vila Franca de Xira, Portugal; 2 Nova Medical School, Universidade Nova de Lisboa, Lisbon, Portugal; 3 Grupo de Investigação e Desenvolvimento em Infeção e Sépsis (GISID), Porto, Portugal; 4 Intensive Care Department, Centro Hospitalar Universitário de S. João, Porto, Portugal; 5 Intensive Care Department, Centro Hospitalar Universitário de Lisboa Norte, Lisbon, Portugal; 6 Intensive Care Department, Centro Hospitalar Trás-os-Montes e Alto Douro, Vila Real, Portugal; 7 Intensive Care Department, Centro Hospitalar Vila Nova de Gaia e Espinho, Vila Nova de Gaia, Portugal; 8 Intensive Care Department, Centro Hospitalar Universitário de Coimbra, Coimbra, Portugal; 9 Intensive Care Department, Centro Hospitalar Universitário do Algarve, Faro, Portugal; 10 Faculdade de Medicina, Universidade do Porto, Porto, Portugal

**Keywords:** Follow-up, Long term, Age, Survival, Mortality, SAPS-II, Standard mortality ratio

## Abstract

**Background:**

The past years have witnessed dramatic changes in the population admitted to the intensive care unit (ICU). Older and sicker patients are now commonly treated in this setting due to the newly available sophisticated life support. However, the short- and long-term benefit of this strategy is scarcely studied.

**Methods:**

The Critically Ill patients’ mortality by age: Long-Term follow-up (CIMbA-LT) was a multicentric, nationwide, retrospective, observational study addressing short- and long-term prognosis of patients admitted to Portuguese multipurpose ICUs, during 4 years, according to their age and disease severity. Patients were followed for two years after ICU admission. The standardized hospital mortality ratio (SMR) was calculated according to the Simplified Acute Physiology Score (SAPS) II and the follow-up risk, for patients discharged alive from the hospital, according to official demographic national data for age and gender. Survival curves were plotted according to age group.

**Results:**

We included 37.118 patients, including 15.8% over 80 years old. The mean SAPS II score was 42.8 ± 19.4. The ICU all-cause mortality was 16.1% and 76% of all patients survive until hospital discharge. The SAPS II score overestimated hospital mortality [SMR at hospital discharge 0.7; 95% confidence interval (CI) 0.63–0.76] but accurately predicted one-year all-cause mortality [1-year SMR 1.01; (95% CI 0.98–1.08)]. Survival curves showed a peak in mortality, during the first 30 days, followed by a much slower survival decline thereafter. Older patients had higher short- and long-term mortality and their hospital SMR was also slightly higher (0.76 vs. 0.69). Patients discharged alive from the hospital had a 1-year relative mortality risk of 6.3; [95% CI 5.8–6.7]. This increased risk was higher for younger patients [21.1; (95% CI 15.1–39.6) vs. 2.4; (95% CI 2.2–2.7) for older patients].

**Conclusions:**

Critically ill patients’ mortality peaked in the first 30 days after ICU admission. Older critically ill patients had higher all-cause mortality, including a higher hospital SMR. A long-term increased relative mortality risk was noted in patients discharged alive from the hospital, but this was more noticeable in younger patients.

**Supplementary Information:**

The online version contains supplementary material available at 10.1186/s13613-023-01102-3.

## Introduction

During the last couple of years changes in the population admitted to the Intensive Care Units (ICU) have been increasingly noted [1], namely with a rise in the mean age and a growing number of comorbidities. Few limitations are now imposed on patients who need invasive treatments or surgery, based solely on old age or even significant frailty, mostly because of the existence of sophisticated treatments and optimal supportive intensive care.

However, it is poorly understood if this benefit, a decrease in hospital mortality of patients admitted to the ICU, is transversal to all age groups. Moreover, it is poorly understood if it persists in the long term and how it is affected by age.

Understanding the prognosis of patients, not only in the short term (ICU and in-hospital mortality) but also in the long term (one or more years of follow-up) is of the utmost importance to provide patients with the relevant information, to design realistic goals of care, to empower patients and their families and to improve satisfaction [2].

Age is strongly associated with prognosis in the critically ill population and several follow-up studies focus on Old and Very Old patients [2–4]. The disease process as well as the prescribed treatment may both jeopardize patients’ health and quality of life. The recovery ability is related to the physiological reserve, which may be more compromised in older and frail patients [5–8]. However, the impact of critical disease on prognosis and quality of life has been well documented for all age groups [4] and admission to the ICU along with invasive treatments may not be the best choice for some patients.

Data regarding short- and long-term prognoses may help to improve doctor-patient communication, especially for older and frail patients, but also for those with higher disease severity or after a long ICU stay (“chronic critically ill disease” [9]). This may facilitate patients, their relatives, and physicians to better define realistic goals of care.

In this large, multicentre study we addressed the short- and long-term prognosis of patients admitted to the ICU, according to their age, gender, type of admission, sepsis on admission, and disease severity.

## Methods

**Fig. 1:**
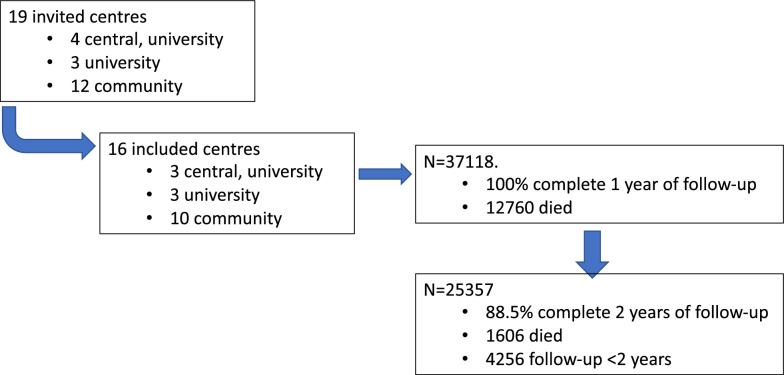
Flowchart of invited and included centres, patients’ mortality, and follow-up

This was a retrospective, multicentre, observational study addressing short- and long-term prognosis of patients admitted to Portuguese multipurpose ICUs. Participation was by invitation only and no financial incentives were provided. Only centres from public hospitals that had a prospective local, electronic database, including demographic and outcome data, were invited to the study. Of the 19 invited centres, 3 declined to participate (Fig. 1).

The study protocol was approved by Hospital Vila Franca de Xira Ethics Committee in 01-10-2020. Informed consent was waived due to the study's retrospective, anonymous nature. The different local Research and Ethics Committees approved the study in all participating centres.

Our primary goal was to measure the one-year mortality rate for every age group and to compare it with the predicted mortality, both according to age and disease severity at ICU admission and according to national epidemiological data. Secondary endpoints included hospital mortality and 2-year all-cause mortality, also for every age group.

All adult patients consecutively admitted to any of the participating study centres during the study 4-year period, (January 2015 to June 2019), for more than 24 h were included. Data were extracted from the administrative database of the participating 16 medical ICUs and were managed locally. After anonymization, all data were collected in a central dedicated database. Data collected at ICU admission included age, gender, the main cause of admission [medical, or either scheduled or non-scheduled (urgent) surgical admission], Simplified Acute Physiology Score (SAPS) II severity score, and presence of sepsis (diagnosed by local criteria). Patients were followed until hospital discharge (either dead or alive) and during the first 2 years of follow-up. The time between ICU admission and death was computed. Each centre was responsible for completing the follow-up of their patients, according to the study protocol. These data were collected from personal contact, Hospital registries, or the National Health Ministry database.

The Standardized Mortality Ratio (SMR) was calculated as the ratio between the observed mortality and the predicted mortality, according to the SAPS II score. This ratio was calculated for the whole population at hospital discharge. An exploratory analysis, using survival data after the first and the second year of follow-up as comparators were performed.

We split our population into 4 commonly evaluated age groups, according to their age at ICU admission: 18–50 years (Adult); ≥ 50–65 years (Senior); ≥ 65–80 years (Old); ≥ 80 years (Very old). Survival curves were plotted for all groups and the log-rank test was used to assess differences between them. We also compared the survival curves according to the admission type and the presence of sepsis on admission. After a visual inspection of the mortality curves, which showed a clear, parallel drop around the 30th day, we split the two years mortality risk into three periods: The first 30 days (after ICU admission); for the 30-day survivors, the interval between the 31st and the 365th day; and for the one-year survivors, the second year of follow-up. The mortality hazard ratio [adjusted for SAPS II score and ICU length of stay (LOS)] was computed for each group.

The excess long-term mortality risk of patients discharged alive from the hospital was calculated as the ratio between the observed mortality in this period and the one predicted for a control group, of the same age and gender, according to official data published for the general Portuguese population [10].

To calculate the relative risk of dying during the follow-up of the critical disease (both the insult and the related ICU admission), we compared the one-year survival of the population discharged alive from the hospital, with that of one virtual group, of the same gender and age, computed according to the official published mortality tables from the Portuguese National Institute of Statistics [10].

This study followed the STROBE checklist for observational studies (https://www.strobe-statement.org/checklists/).

### Statistical analysis

General descriptive statistics were used. Continuous variables were reported as mean ± standard deviation, or median [25–75 interquartile range], according to data distribution. Categorical variables were reported as counts (percentage).

Continuous baseline demographics and clinical data were compared with the Student *T* test or Mann–Whitney *U* test, according to data distribution. Categorical data were compared with the Chi-square test. ANOVA was used to compare the SAPS II score between age groups (with and without age points).

The Cox proportional hazard was used to compare the mortality risk of the different groups of patients. Hazards ratios, adjusted for SAPS II score and ICU LOS, were computed along with the 95% confidence interval (CI).

To account for a potential centre effect, we developed a multiple logistic regression analysis with one-year all-cause mortality as the dependent variable. The admitting centre was forced into the model (along with gender, SAPS II, type of admission, age group, and the presence of sepsis on admission).

Statistical analysis was performed using IBM SPSS Statistics v.25.0 (IBM, Somers, NY, USA). All statistics were two tailed, and the significance level was defined as *p* < 0.05.

## Results

We included 37,118 patients (60.1% males), from 16 different centres, covering roughly 65% of all available beds from Portuguese ICUs. Only 3 other invited centres declined to participate (Fig. 1). We included 6 university hospitals (3 of them were also central hospitals), and 10 community hospitals (Fig. 1). The patients’ mean age was 64 ± 16.4 years (male 63.0 ± 16.1, female 65.6 ± 16.8 years). The mean SAPS II score was 42.8 ± 19.4, with a normal distribution. There were some differences in SAPS II score according to age groups (Table 1), even after discarding the age points (ANOVA *p* < 0.001). A medical cause for admission was noted in 63.3% of patients, whilst only 11.8% were admitted after elective surgery. Sepsis on admission to the ICU stay was identified in 31.3% of patients.

**Table 1:** Patients’ characteristics on ICU admission

	*N*	Male (%)	SAPSII (mean ± SD)		Type of admission (%)	LOS		Sepsis (%)
			Total^a^	Without age points^a^		ICU (median [IQR])	Hospital^b^ (median [IQR])	
18–50 Adult	7632	62.6	32.43 ± 18.16	28.59 ± 17.9	M 59.4 US 30.1 S 10.6	4 [2–9.1]	8.4 [4–20.2]	24.6
≥ 50–65 Senior	9933	65.6	40.13 ± 18.62	30.48 ± 18.42	M 62.5 US 23.2 S 14.3	4.2 [2–9.9]	10 [5–22.5]	29.3
≥ 65–80 Old	13,667	58.8	47.20 ± 18.48	33.03 ± 18.34	M 64 US 23.2 S 12.7	4.3 [2–9]	10.2 [5.2–22]	34.6
≥ 80 Very-old	5886	50.1	50.71 ± 17.29	32.74 ± 17.22	M 65.9 US 26.9 S 7.2	4 [2–7.6]	9.4 [5–19]	35.4
Total	37,118	60.1	42.83 ± 19.36	31.39 ± 18.18	M 63 US 25.2 S 11.8	4 [2–9]	10 [5–21]	31.3

*ICU* intensive care unit, *LOS* length of stay, *SAPS* simplified acute physiology score, *SD* standard deviation, *IQR* interquartile range, Type of admission (*M* medical, *US* unscheduled surgery, *S* surgery)

^a^ANOVA *p* < 0.001;

^b^after ICU discharge

The median ICU LOS was 4 [2–9] days including 146 patients (0.4%) with an ICU LOS of more than 60 days (Table 1).

There were some differences between centres: the mean age ranged between 60.6 ± 15.7 and 70.4 ± 15.2 and the SAPS II score ranged between 35.99 ± 15.89 and 52.51 ± 22.41. In the multiple logistic regression model, the one-year all-cause mortality remained associated with age group [young reference; senior: 1.43 (1.32–1.56); old: 1.85 (1.71–2.0); very old: 2.78 (2.54–3.01), p < 0.001]—Additional file 1.

Patients older than 80 years account for almost 15.8% of all admissions (Table 1). Patients discharged alive from the ICU (*N* = 31,136) had a median hospital LOS (after ICU discharge) of 10 [5–21] days, including 12.2 [4–31.5] days for nonsurvivors.

### Mortality according to age group and disease severity

**Fig. 2:**
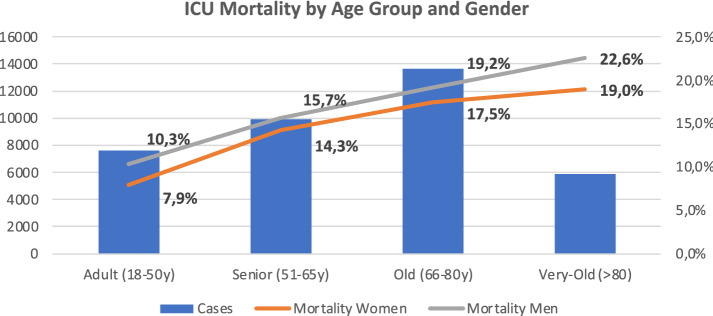
ICU Mortality by age group and gender. The bars show the number of cases by age group. Lines show mortality (%) by age group and gender

The total ICU all-cause mortality was 16.1%. This increased with age and was slightly higher in the male gender (*p* < 0.001)—Fig. 2. Of the 31,136 patients discharged alive from the ICU, 9.4% died in the hospital. This ward mortality was higher in males (10.1% vs. 8.5%; *p* < 0.001). Not surprisingly, SAPS II was much higher in ICU non-survivors (61.2 ± 18.1 vs. 39.3 ± 17.5, *p* < 0.001) as well as in in-Hospital non-survivors (57.9 ± 18.2 vs. 38.1 ± 17.1, *p* < 0.001).

**Fig. 3:**
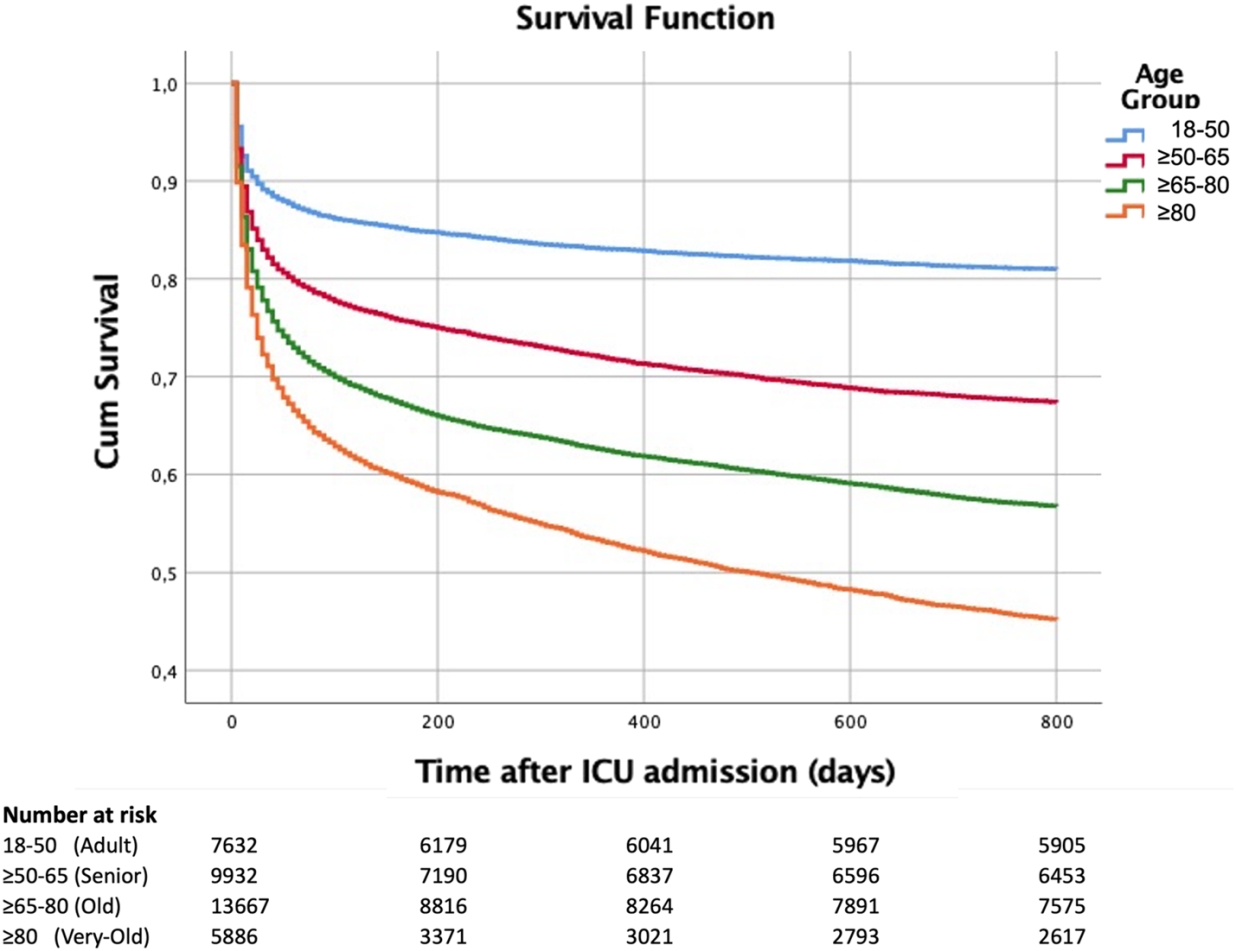
Survival curves by age group. Survival during the 2 years of follow-up after Intensive Care Unit admission by Age Group; Log Rank test *p* < 0.001

Overall, 76% of patients that stayed more than 24 h in the ICU were discharged alive from the hospital. During follow-up, the mortality of the hospital survivors was 14.9% during the first year, rising to a total of 20.5% after two years. In Fig. 3, the survival curves according to the age group are presented. Early high mortality (between days 1 and 30) was noted. Afterward, there was a long-term declining survival from day 31 onwards [more pronounced in older patients, even after adjustment for SAPS II score and ICU LOS (Table 2)]. The median time between hospital discharge and death (for those that died during the first year of follow-up) was 89 [161] days.

**Table 2:** Risk of mortality during the first month, between days 31^st^ and 365^th^ and the second year after ICU admission

Variable	First-Month mortality (*n* = 37,118)			First-year mortality (N 30045)			Second-year mortality (N 20102)		
	No. (%)			No. (%)			No. (%)		
	Alive (*n* = 30,045)	Dead (*n* = 7073)	aHR (95% CI)	Alive (*n* = 24,358)	Dead (*n* = 5687)	aHR (95% CI)	Alive (*n* = 18,496)	Dead (*n* = 1606)	aHR (95% CI)
Age									
18–50 (Adult)	6831 (89.5)	801 (10.5)	Reference	6057 (88.7)	774 (11.3)	Reference	4776 (97.2)	136 (2.8)	Reference
≥ 50–65 (Senior)	8280 (83.4)	1653 (16.6)	1.18 (1.09–1.29)	6888 (83.2)	1392 (16.8)	1.48 (1.35–1.61)	5285 (93.1)	389 (6.9)	2.61 (2.14–3.17)
≥ 65–80 (Old)	10,672 (78.1)	2995 (21.9)	1.19 (1.1–1.28)	8342 (78.2)	2330 (21.8)	1.88 (1.73–2.05)	6258 (90.2)	683 (9.8)	3.9 (3.23–4.71)
≥ 80 (Very-old)	4262 (72.4)	1624 (27.6)	1.3 (1.19–1.42)	3071 (72.1)	1191 (27.9)	2.42 (2.21–2.66)	2177 (84.5)	398 (15.5)	6.48 (5.3–7.94)
Gender									
Male	17,857 (80.1)	4437 (19.9)	Reference	14,352 (80.4)	3505 (19.6)	Reference	10,875 (91.7)	983 (8.3)	Reference
Female	12,188 (82.2)	2636 (17.8)	0.88 (0.84–0.92)	10,006 (82.1)	2182 (17.9)	0. 90 (0.86–0.95)	7621 (92.4)	623 (7.6)	0.91 (0.82–1.01)
Admission type									
Medical	18,348 (78.5)	5022 (21.5)	Reference	14,715 (80.2)	3633 (19.8)	Reference	11,091 (91.9)	982 (8.1)	Reference
Schedule surgery	4087 (93.1)	302 (6.9)	0.53 (0.47–0.6)	3408 (83.4)	679 (16.6)	0.99 (0.91–1.08)	2488 (88.7)	317 (11.3)	1.61 (1.41–1.84)
Unscheduled Surgery	7610 (81.3)	1749 (18.7)	0.86 (0.81–0.91)	6235 (81.9)	1375 (18.1)	0. 88 (0.83–0.94)	4917 (94.1)	307 (5.9)	0.71 (0.63–0.81)
Infection									
No Sepsis	20,946 (83.2)	4231 (16.8)	Reference	17,354 (82.9)	3692 (17.1)	Reference	13,139 (92.2)	1108 (7.8)	Reference
Sepsis	8691 (75.9)	2757 (24.1)	1.13 (1.08–1.19)	6662 (76.7)	2029 (23.3)	1.29 (1.22–1.36)	5085 (91.5)	474 (8.5)	1.06 (0.95–1.18)

*aHR* Hazards ratio adjusted for simplified acute physiology score II and intensive care unit length of stay, *CI* confidence interval

^a^In 493 patients there was no information regarding sepsis

^b^4256 patients with incomplete two-year follow-up were excluded

In Figure 2 (Additional file 2), we present the short- and long-term relative risk of death according to age group, type of admission, and sepsis. The older population and those presenting with sepsis had a higher mortality rate. Patients submitted to elective surgery had initially lower mortality, but this difference faded during the follow-up (Table 2), and for the 30-day survivors, long-term mortality was higher for this group than for patients submitted to urgent, unscheduled surgery (Additional file 2).

We computed the SMR at hospital discharge (as previously described [11]), according to the SAPS II score at ICU admission. The results are presented in Table 3. Again, the SMR was higher in the very-old group (0.76 vs. 0.69). We repeated these calculations using mortality data after one year of follow-up (again using the SAPS II predicted mortality as a comparator) and found a value close to one for every age sub-group, slightly higher in the very old (Table 3).

**Table 3:** Mortality rate by Age Group according to their risk at ICU admission

Age group	*N*	Predicted Hospital Mortality^a^ (%)	ICU Mortality (%)	Hospital Mortality (%)	Hospital SMR^b^	1 Year SMR^b^	2-Years SMR^b^
18–50 Adult	7632	20.7	9.4	13.1	0.63	0.99	1.07
≥ 50–65 Senior	9933	30.2	15.2	21.4	0.71	1.01	1.14
≥ 65–80 Old	13,667	39.6	18.5	27.9	0.70	0.98	1.11
≥ 80 Very-Old	5886	44.4	20.8	33.5	0.76	1.08	1.24
Total	37,118	34	16.1	24	0.71	1.01	1.14

Evaluation of mortality relative risk at hospital discharge, one-year, and two-year follow-up (in the two years of follow-up only the 29,661 patients with complete data were considered)

*ICU* intensive care unit, *SMR* standard mortality ratio

^a^Hospital mortality predicted according to the Simplified Acute Physiology Score II

^b^SMR calculated from mortality data at hospital discharge, one and two years of follow-up (see text for details)

### Excess mortality after hospital discharge

**Fig. 4:**
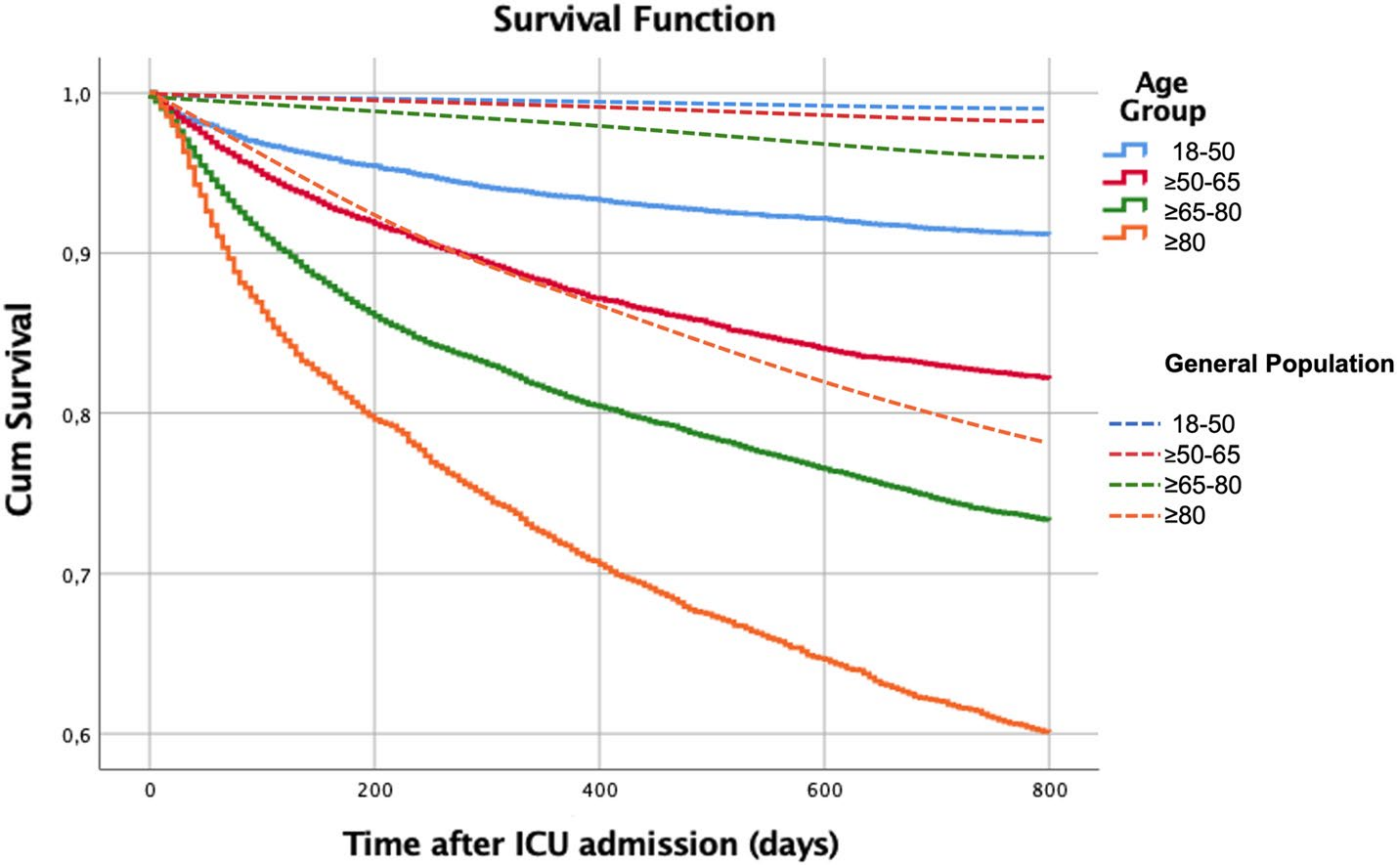
Survival curves by age group of patients and of the general population. Only patients discharged alive from the Hospital were included. Continuous line—Patients; dashed line—General population (of the same gender and age).

The long-term (after day 30) absolute and relative mortality risks according to age are presented in Table 4 and Fig. 4, for every sub-group.

**Table 4:** Long-term excess mortality according to age group

Age groups (years)	Gender	*N*	Observed one-year mortality	Relative risk	95% Confidence interval	*p*-value	
18–50 Adult	Male	4092	478	21.7	(14.2–33.2)		< 0.001^**^
	Female	2538	261	20.1	(11.5–35)	0.079^*^	
	Total	6630	739	21.1	(15.1–39.6)		
≥ 50–65 Senior	Male	5060	857	15.3	(11.7–20)		
	Female	2746	383	22.5	(13.9–36.5)	0.001^*^	
	Total	7806	1240	17.0	(13.4–21.5)		
≥ 65–80 Old	Male	5696	1268	7.2	(6.2–8.4)		
	Female	4158	817	10.6	(8.4–13.3)	0.002^*^	
	Total	9854	2085	8.2	(7.3–9.4)		
≥ 80 Very-old	Male	1850	598	2.4	(2.1–2.7)		
	Female	2062	564	2.5	(2.2–2.9)	0.001^*^	
	Total	3912	1162	2.4	(2.2–2.7)		
Total	Male	16,698	3201	6.3	(5.8–6.9)		
	Female	11,504	2025	6.1	(5.5–6.8)	0.001^*^	
	Total	28,202	5226	6.3	(5.8–6.7)		

Only patients discharged alive from the Hospital were included. Observed mortality refers to the absolute number of deaths that occurred; Relative risk according to the estimated mortality for a virtual population of the same age and gender, according to Portuguese data published by the Portuguese National Institute of Statistics, 2020 [10]

Chi-square test for:

^*^Differences between males and females

^**^Differences between age groups

The relative mortality risk for each age stratum, compared to a similar population of the same age and gender, virtual mortality risk [10], is presented in Table 4. Of note, there was an increase of 6.3 (95% CI 5.8–6.7) times in the risk of mortality during the first-year post-hospital discharge. This excess mortality relative risk was lower in the older population, although their absolute mortality was much higher (Table 4).

## Discussion

The CIMbA-LT study provides data on critically ill patients' short- and long-term prognosis. An early, high, mortality risk during the first 30 days after ICU admission, was unveiled, probably related to the acute disease. This decreased sharply thereafter across all age groups. The SMR at hospital discharge (according to the SAPS II predicted mortality) for the whole population was 0.7. The SMR was slightly higher at hospital discharge in the older population (Table 3). An exploratory analysis, to evaluate the SAPS II score performance in addressing the 1-year and 2-year mortality, was performed, showing an SMR around 1 after one year and around 1.14 at two years, again both slightly higher in the older population. We believe that this topic deserves further studies to address a potential role for SAPS II unveiling one-year after ICU admission prognosis of patients receiving modern intensive care.

The SAPS II score was first introduced to help to predict hospital mortality (of critically ill patients) [11]. In an interesting study, authors noted that SMR (that is severity adjusted hospital mortality) increased with age, especially in patients with less burden of intensive care interventions [12]. On the opposite, in our study, this increase was not so striking, which may be related to the higher severity of our cohort.

The decision to admit a patient to the ICU should be based on the perceived benefit of ICU treatment and its prognosis. This may be especially challenging when dealing with the very old [1, 5] or the frail patient [6, 8, 13], in whom chronic comorbidities may jeopardize ICU potential benefits, especially as it is well-known that these episodes are associated with a decline in the quality of life [14].

In our study, we unveiled the usefulness of the SAPS II score also to predict long-term mortality [2, 15]. This may be especially important since older patients often have very high absolute mortality at every time point, reaching rates over 50% [2, 4]. Nevertheless, families often have excessively optimistic perspectives on the patient’s prognosis [16]. Consequently, both overenthusiastic and excessively pessimistic views may both be detrimental to older patient management. In another study, the survival risk associated with critical illness seems to be related to age and gender itself, and, after adjustment for these two factors, the impact of intensive care admission on survival and quality of life was similar to the one in younger patients [4]. This highlights the importance of individualization of ICU admission, according to patient health status and own values, not only age or disease severity.

When in doubt, an ICU trial [17] has been proposed for oncological patients. This means treating the patient as much as possible for a short period, but being prompt to withdraw therapy to avoid dysthanasia, in those not responding to therapy. The same may be applied to this group of older patients. This may also help facilitate communication and design realistic care goals for patients and their relatives [18].

Even after hospital discharge, a very high relative risk of long-term mortality was noted in our population, over 6 times higher than the general population. Similar findings were noted in another large study from France, a 6.64 (95% CI 6.61–6.73) increase in the relative risk of death during the first year after hospital discharge. This was noted to be independent of organ support during ICU stay and, similarly to our study, decreased with increasing age [19]. Older patients often present comorbidities and other risk factors that probably superimpose, and their absolute mortality risk progressively resembles that of a control population. The old and the frail patient probably will require a different approach and their management imposes different challenges [18, 20]. On the contrary, younger patients may need more hospital resources [21]. These differences may be more striking in patients admitted to the ICU with a respiratory infection or sepsis [22–24].

Further studies are needed to understand the long-term determinants of ICU outcomes and how to influence them [25].

## Limitations

This was a retrospective study; consequently, unintended bias may have been present. Nevertheless, the large sample we included may have helped minimize this risk. Moreover, we did not collect data on frailty, or the therapeutic limitations policies and the same may have influenced our results.

We also did not evaluate therapeutic interventions (including the use of organ support therapies, especially invasive mechanical ventilation, renal replacement therapy, and vasopressors), comorbidities, and the precise reason for admission. Consequently, we cannot exclude some group overrepresentation.

Furthermore, the need for organ support, and other variables such as functional status, frailty, and comorbidities, may all have influenced short- and long-term mortality.

Also, local differences in admission policies and the use of the SAPS II score (which has been shown to have imperfect calibration across centres [26]) may have influenced our results.

Changes in the sepsis definition occurred during the study period. As we accepted local criteria for the diagnosis of sepsis, heterogeneity may have occurred and limited the interpretation of this variable.

Finally, we did not collect data on patients’ quality of life after hospital discharge, neither on hospital readmissions nor causes of death.

National registries to study the influence of these different factors on prognosis should be promoted.

## Conclusion

We unveiled a high mortality risk after ICU admission that significantly decreases by day 30. Older patients had a higher hospital mortality risk than younger ones, with a slightly higher SMR. A 6.3 times excess relative risk mortality after ICU discharge was noted for hospital survivors, and this was higher in younger patients.

The SAPS II severity score overestimated hospital mortality but accurately predicted the one-year mortality rate.

## Supplementary Information

**Additional file 1: Table S1. **Multiple logistic regression analysis with one-year all-cause mortality as the dependent variable. The admitting centre was forced into the model.

**Additional file 2: Fig. S1.** Kaplan–Meier survival curves for the first month and for the 31st day to 2 years after Intensive Care Unit admission, according to age group, admission type, and sepsis. *Only 30-day survivors were included in the right panels.

## Data Availability

The datasets used and/or analyzed during the current study are available from the corresponding author on reasonable request.

## Abbreviations

ICU: Intensive care unit
CIMBA-LT: Critically ill patients’ mortality by age: long-term follow-up
SMR: Standard mortality ratio
SAPS II: Simplified acute physiology score
CI: Confidence interval
LOS: Length of stay
