# Supplementary material for: Critically ill patient mortality by age: long-term follow-up (CIMbA-LT)
Source: Ann Intensive Care. 2023 Feb 11;13:7. doi: 10.1186/s13613-023-01102-3 (PMC9918627; doi:10.1186/s13613-023-01102-3)
Supplement: Supplementary file 1 — Additional file 1: Table S1. Multiple logistic regression analysis with one-year all-cause mortality as the dependent variable. The admitting centre was forced into the model. [file 13613_2023_1102_MOESM1_ESM.docx]

**Additional File**

Table S1 –Risk factors for one-year all-cause mortality including a “center effect”

|  | p-value | Odds Ratio | 95% Confidence Interval | |
| --- | --- | --- | --- | --- |
|  |  |  | Lower | Higher |
| Center 1 |  | 1 [reference] |  |  |
| Center 2 | <0.001 | 0.71 | 0.61 | 0.83 |
| Center 3 | <0.001 | 0.30 | 0.24 | 0.37 |
| Center 4 | <0.001 | 0.50 | 0.41 | 0.60 |
| Center 5 | 0.16 | 1.13 | 0.96 | 1.33 |
| Center 6 | 0.041 | 0.86 | 0.74 | 0.99 |
| Center 7 | <0.001 | 0.71 | 0.62 | 0.81 |
| Center 8 | <0.001 | 0.10 | 0.08 | 0.12 |
| Center 9 | 0.26 | 0.93 | 0.81 | 1.06 |
| Center 10 | 0.021 | 1.20 | 1.03 | 1.40 |
| Center 11 | 0.003 | 1.26 | 1.08 | 1.46 |
| Center 12 | <0.001 | 0.07 | 0.06 | 0.10 |
| Center 13 | <0.001 | 0.71 | 0.63 | 0.80 |
| Center 14 | 0.21 | 0.90 | 0.76 | 1.06 |
| Center 15 | <0.001 | 0.71 | 0.62 | 0.82 |
| Center 16 | <0.001 | 0.53 | 0.46 | 0.61 |
|  |  |  |  |  |
| Gender female |  | 1 [reference] |  |  |
| Gender male | <0.001 | 0.81 | 0.77 | 0.86 |
|  |  |  |  |  |
| SAPS II Score | <0.001 | 1.04 | 1.04 | 1.04 |
|  |  |  |  |  |
| Age Group (>50) |  | 1 [reference] |  |  |
| Age Group (≥50-65) | <0.001 | 1.43 | 1.32 | 1.56 |
| Age Group (≥65-80) | <0.001 | 1.85 | 1.71 | 2.00 |
| Age Group (≥80) | <0.001 | 2.78 | 2.54 | 3.04 |
|  |  |  |  |  |
| No Sepsis |  | 1 [reference] |  |  |
| Sepsis | <0.001 | 1.10 | 1.05 | 1.17 |
|  |  |  |  |  |
| Admission Type (Scheduled Surgery) | | 1 [reference] |  |  |
| Admission Type (urgent Surgery) | 0.27 | 1.06 | 0.96 | 1.17 |
| Admission Type (medical) | <0.001 | 1.20 | 1.10 | 1.32 |
