# Supplementary figures and images for: Critically ill patient mortality by age: long-term follow-up (CIMbA-LT)
Source: Ann Intensive Care. 2023 Feb 11;13:7. doi: 10.1186/s13613-023-01102-3 (PMC9918627; doi:10.1186/s13613-023-01102-3)

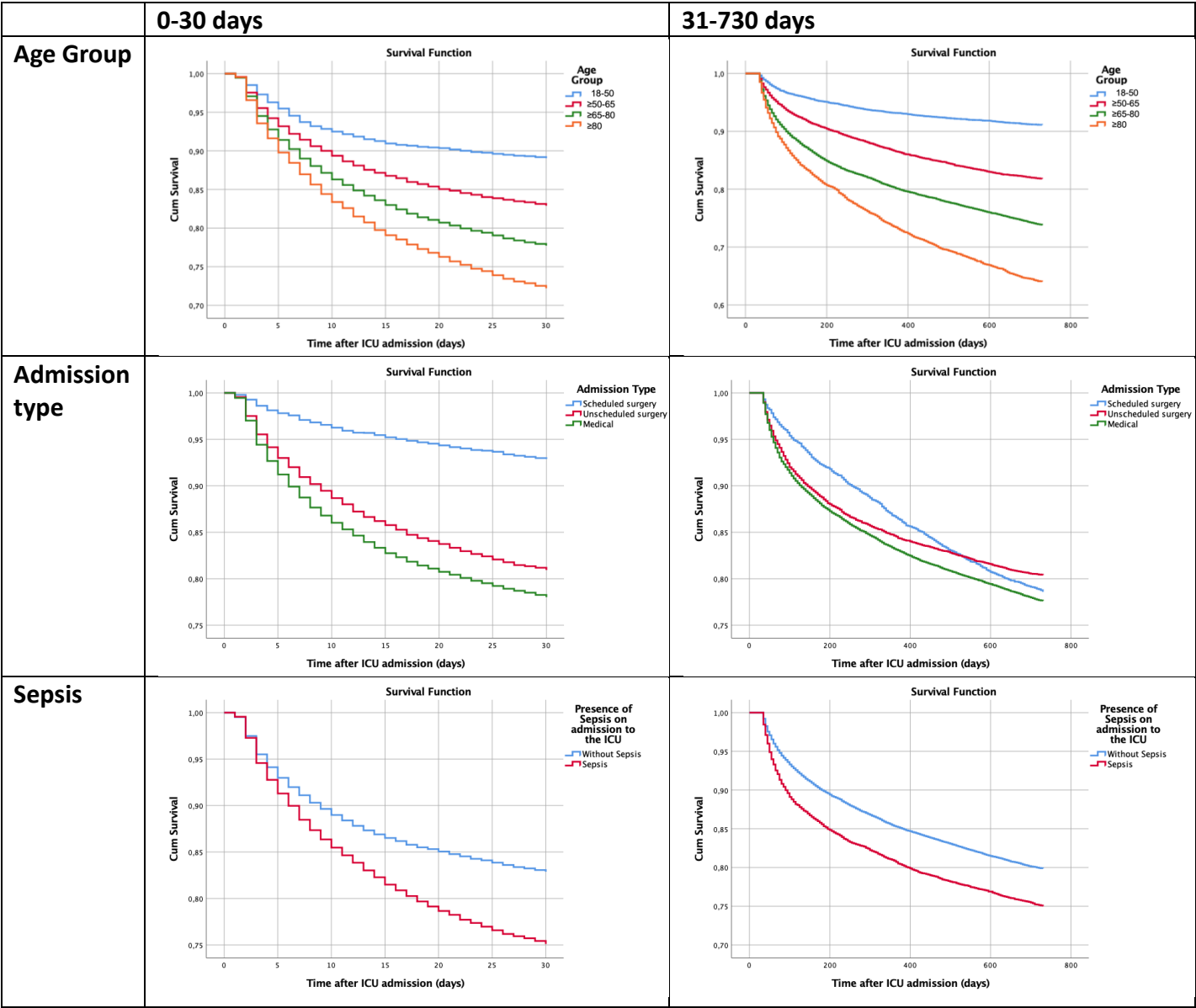

Supplement: Supplementary file 2 — Additional file 2: Fig. S1. Kaplan–Meier survival curves for the first month and for the 31st day to 2 years after Intensive Care Unit admission, according to age group, admission type, and sepsis. *Only 30-day survivors were included in the right panels. [file 13613_2023_1102_MOESM2_ESM.pdf]
